# Pre-dispositional constitution and plastic disposition: toward a more adequate descriptive framework for the notions of habits, learning and plasticity

**DOI:** 10.3389/fnhum.2014.00341

**Published:** 2014-05-27

**Authors:** Francisco Güell

**Affiliations:** Mind–Brain Group, Institute for Culture and Society, Universidad de NavarraPamplona, Spain

**Keywords:** habits, motor cognition, associative learning, mirror neurons, schizophrenia, autistic disorder, neural plasticity, neural migration

Behavioral studies and neurobiological models of mental illnesses can be used to inform theories of mind and action. In this paper I use specific aspects of some paradigmatic cases in order to establish what I consider to be a useful distinction for the analysis of human action and, more specifically, the delimitation of habitual action.

Patient SM is a well-known case of Urbach-Wiethe disease—one of only 300 cases reported in the reference literature—that was submitted to a decade of investigations (Adolphs et al., 1994, 2005). This syndrome, also known as lipoid proteinosis, produces dermatological lesions as well as calcifications in regions of the brain, often affecting the amygdaloid region (Siebert et al., 2003; Bahadir et al., 2006). While her basic perception, memory, and language skills are essentially normal, SM has nearly complete bilateral destruction of the amygdala, and her social behavior is indiscriminately trusting and friendly (Adolphs et al., 1994). Ten years of research showed an intriguing impairment in her ability to recognize fear in facial expressions, due to a lack of spontaneous fixation on the eyes when viewing faces (Adolphs et al., 2005). The research showed that in control patients, spontaneous fixation is directed principally to the eyes and the mouth, tracking the regions of the face that allow one to distinguish facial expressions. However, patient SM spontaneously focused on the nose, thereby missing necessary information for judging emotions. What is so interesting to point out is that when given explicit instructions (“look at the eyes of this person”) SM had no problem focusing on the eyes and recognizing emotions but, surprisingly, after a decade of treatment, SM was not able to learn the habit of looking at the eyes of the face spontaneously.

Another case of interest for action theory can be found in studies of subjects in the autistic spectrum (Klin et al., 2003; Boria et al., 2009; Gallese, 2009; Kana et al., 2014), particularly studies of deficits in the functioning of the “mirror mechanism” (Antonietti, 2013). This deficit appears related to other deficits such as atypical visual processing and encoding of social stimuli, as well as imitative behavior and the ability to share attention (for review, see Gallese et al., 2013). Atypical brain development has been identified in cases of autism; specifically, the neural organization in areas involved in social cognition, facial expression, and facial recognition, as well as in areas associated with the mirror mechanism, appears to be related to the functional architecture that characterizes the atypical development of the autistic spectrum (Cauda et al., 2011; Gallese et al., 2013).

Another paradigmatic case that is informative for theories of mind-based action is schizophrenia (Synofzik et al., 2010; Leube et al., 2012; Mausbach et al., 2013). Multiple investigations suggest that schizophrenia and other neuropsychiatric disorders are associated with deficits in mirror neurons (Enticott et al., 2008; Mehta et al., 2013) and with interneuron dysfunction (Marin, 2012). For now, let us focus on the second dysfunction. Interneurons regulate the activity of pyramidal cells, largely through inhibitory mechanisms, and one of the functions of pyramidal cells is to maintain cerebral patterns associated with perception and memory. Migration of the interneurons during the development of the nervous system is fundamental to this function, as it determines the final positioning of the neurons, thereby establishing the basis for correct wiring of neural circuitry (Marín, 2013). It has been demonstrated that schizophrenics possess mutations in certain genes that affect the migration of the intercortical neurons during embryonic development (Valiente and Marin, 2010). In addition, for decades we have known that there is a correlation between schizophrenia and the alteration of visual perception and eye movements. In fact, just recently it has been shown that simple tests for the detection of abnormal eye movements can discriminate cases of schizophrenia from controls with exceptional accuracy (Benson et al., 2012).

Of the genes that are involved in the disrupted tangential migration of cortical neurons, NRG1, ERBB4, GRIN1, DISC1, and DTNBP1 (Marin, 2012), four of them are involved in the expression of molecules related with visual structures, and one of them is related to early visual processing: NRG1 is expressed in the cornea (Brown et al., 2004) and one of its mutations (rs3924999) affects spatial accuracy on the anti-saccade (AS) task (Schmechtig et al., 2010) and is associated with auditory P300 in schizophrenia (Kang et al., 2012); ERBB4 (especially, rs7598440) is associated with 8 endophenotypes, including AS abnormality and smooth pursuit eye movement (Greenwood et al., 2011; Baea et al., 2012); two N-methyl-D-aspartate receptor subunits (NMDARs) encoded by the gene GRIN1 belong to the ionotropic glutamate receptors, which play key roles in neuronal communication in the retina (Fana et al., 2013). DTNBP1 affects the expression of Dysbindin (Benson et al., 2001), a protein whose deficit is associated with early visual deficits in schizophrenia (Donohoea et al., 2008).

Now, to understand the import of these and other cases, it is helpful to introduce a distinction between *constitutional pre-disposition* and *dispositional plasticity*. The neural architecture of SM pre-disposes her to look at faces in a certain way that resists training; likewise it has been shown that the constitutional pre-disposition of schizophrenia does not permit modification through the acquirement of new habits, and the same can be said for autistic individuals. In all of these cases, an atypical neural organization constitutionally pre-disposes the subject to perceive the world in a specific way. For example, it seems that schizophrenics cannot perceive the kinds of optical illusions normally perceived by healthy individuals. It is important to note that this pre-disposition does not need to be understood in terms of genetic determinism. Instead, studies point toward changes at the epigenetic level that affect neuronal plasticity (Fagiolini et al., 2009; Baker-Andresen et al., 2013), and there is an increasing number of examples of post-natal experiences that are affected at this level (Woldemichael et al., 2014). In addition, environmental factors play a crucial role in the formation of constitutional pre-disposition. For example, the lack of interneuronal migration is also caused by fetal exposure to cocaine (Valiente and Marin, 2010), and it has been demonstrated that individuals possessing susceptibility alleles in genes, such as DISC1, express psychiatric phenotypes only when these genetic variants occur in a propitious genome and when certain environmental pre-natal factors come into play (Abazyan et al., 2012).

Now let us turn to *dispositional plasticity*. This refers to the plastic dimension of the organic substrate, a plasticity that makes possible the modulation and function of biological structures. Activity and environmental stimuli continually modify the disposition of the subject, permitting the subject to obtain, inter alia, a certain tone of skin, to develop muscles or to “perfect” the organism on the most basic motor level through the repetition of a task. However, this perfection or specialization can also occur at the perceptual level (such as in the case of an oenophile) or at the level of higher functions (e.g., enhanced memory capacity). Habits, from this perspective, can be considered as actions that regulate the subject's disposition so as to facilitate a task and make others possible. In short, habits, and generally the repetition of tasks, adjust dispositions to act, and they do this thanks to the plastic character of the organic substrate.

The relative incapacity to regulate the constitutional pre-disposition (once consolidated) does not mean that there is no effective treatment for a subject with a specific constitutional pre-disposition. It is well known that many mental diseases can be treated but, according to the model presented here, such treatments do not modify the constitutional pre-disposition. Instead, what treatments do is compensate for the deficits of a certain constitution (for example, by supplying a neurotransmitter) or establish behavioral strategies that minimize effects on the subject's behavior. For instance, note that when SM looks at the eyes of a person because she is asked to do so, she is not modifying her pre-disposition; she is simply fixing her gaze on a certain point voluntarily, just as she would if she were to read this article. However, the fact that the constitutional pre-disposition cannot be regulated (regulation occurs only at the level of dispositional plasticity) does not mean that it cannot be damaged: continuous consumption of drugs can affect dispositional plasticity and, sooner or later (depending on the constitutional pre-disposition), damage the constitutional level as well.

The proposed distinction may be useful for explaining the risk factors related to cancer, as the constitutional pre-dispositions for developing cancer are varied (indeed, we need to keep in mind that there are as many constitutional pre-dispositions as there are subjects). A constitutional pre-disposition for cancer does not necessarily mean that the subject is going to develop cancer; at the same time, the role of dispositional plasticity helps us to understand the importance of certain external factors which, by affecting the plasticity level, can function as the “trigger” for the appearance of cancer. The concept of constitutional pre-disposition also offers theoretical support for evidence that some ethnic groups are particularly vulnerable to certain diseases (Helgadottir et al., 2006; Ng et al., 2012) and may explain differences of organic reaction to certain therapies or drug use as a function of ethnicity (Marsha et al., 1999; Ono et al., 2013).

Let us consider a further example that shows how the proposed distinction can help to frame current debates over the genetic basis of behavior. Going back to the topic of mirror neurons, there is some debate as to whether the associated neural network is genetically inherited (innate) or if it is the product of associative learning. According to the terms just introduced, this choice between genes and learning is oversimplified; in addition to the genetic dimension and associative learning, we should also keep in mind the epigenetic dimension and the importance of environmental factors. Indeed, the importance of epigenetics has now been well established from the “evo-devo” standpoint (Ferrari et al., 2013). To address this added complexity, we can formulate the question more precisely as follows: does associative learning change the constitutional disposition or act on a level of plasticity, regulating the disposition of the subject?

In conclusion, I believe that the distinction between constitutional pre-disposition and dispositional plasticity offers a conceptual framework that can help place theories of mind and action into its developmental context, throw light on current debates, and offer an interpretative key for results arising from research. This distinction allows us to place the notion of habit within the broadest context of human action and thereby better understand its scope. In closing, it is important to clarify that the statement that some aspects of human behavior cannot be changed should not be taken as a deterministic argument against human freedom. It is merely an expression of the universal and widely recognized experience of human limitations.

## Conflict of interest statement

The author declares that the research was conducted in the absence of any commercial or financial relationships that could be construed as a potential conflict of interest.
